# Acknowledgment to the Reviewers of *Journal of Xenobiotics* in 2022

**DOI:** 10.3390/jox13010004

**Published:** 2023-01-17

**Authors:** Journal of Xenobiotics Editorial Office

**Affiliations:** MDPI AG, St. Alban-Anlage 66, 4052 Basel, Switzerland

High-quality academic publishing is built on rigorous peer review. *Journal of Xenobiotics* was able to uphold its high standards for published papers due to the outstanding efforts of our reviewers. Thanks to the efforts of our reviewers in 2022, the median time to first decision was 18 days and the median time to publication was 63 days. Regardless of whether the articles they examined were ultimately published, the editors would like to express their appreciation and thank the following reviewers for the time and dedication that they have shown *Journal of Xenobiotics*:
Abazar GhorbaniLars Petter JordheimAbdou Azaque ZouréLaura BulgariuAftab AlamLeonid V. PerelomovAgnieszka StawarskaLouis Kuoping ChaoAicha MassraliLucas D. DiasAlexey K. PiskunovMaciej DzikućAlina PaunescuMaciej MalinowskiAmit KumarMaliheh MoradzadehAna LuzioMaria KoufakiAndrea SpallarossaMarta De ZottiBernardo Brito PalmaMatej StuhecCarina LadeiraMehrdad KhatamiChaoqi ChenMichał AntoszczakChristoph WenzelMohamed A. M. El GendyCristina Mihaela GhiciucMonika BilCulleré LauraNatalia AbramenkoDavid A. MacLeodNathalie Zucchini-PascalEdward FreemanNikiforos AlygizakisFurong TianNorman S. AllenGaetano SantulliPawel KafarskiGiuseppe SuariaPeter PolčicHeli KangasRoger A. LaineHiroshi TakemoriRosa Herráez-HernándezIoannis RoussisRosaria ScudieroIrene Gomes Câmara CamachoStefan LyerJames K. H. FangSubramanian SundarrajanJoanna GiebultowiczSubrata BanikJoanna SenderSudeshna SahaJoel EllwangerTomasz W. KaminskiJustyna KowalskaTran Dang XuanKamil Krzysztof RomanVasil AtanasovKamlesh BhopaleVittorio CalabreseKarla Oyuky Juarez-MorenoWen-Tsong HsiehKrystyna Pierzchała-KoziecYue ZhangLaleh Yerushalmi



